# Antioxidative and Cardioprotective Properties of Anthocyanins from Defatted Dabai Extracts

**DOI:** 10.1155/2013/434057

**Published:** 2013-12-04

**Authors:** Hock Eng Khoo, Azrina Azlan, M. Halid Nurulhuda, Amin Ismail, Faridah Abas, Muhajir Hamid, Suri Roowi

**Affiliations:** ^1^Department of Nutrition and Dietetics, Faculty of Medicine and Health Sciences, Universiti Putra Malaysia (UPM), 43400 Serdang, Selangor, Malaysia; ^2^Laboratory of Halal Science Research, Halal Products Research Institute, Universiti Putra Malaysia, 43400 Serdang, Selangor, Malaysia; ^3^Laboratory of Natural Products, Institute of Bioscience, Universiti Putra Malaysia (UPM), 43400 Serdang, Selangor, Malaysia; ^4^Department of Microbiology, Faculty of Biotechnology and Biomolecular Sciences, Universiti Putra Malaysia (UPM), 43400 Serdang, Selangor, Malaysia; ^5^Food Technology Research Centre, MARDI Headquarters, Serdang, P.O. Box 12301, 50774 Kuala Lumpur, Malaysia

## Abstract

This study aimed to determine anthocyanins and their antioxidative and cardioprotective properties in defatted dabai parts. Anthocyanins in crude extracts and extract fractions of defatted dabai peel and pericarp were quantified using UHPLC, while their antioxidant capacity and oxidative stress inhibition ability were evaluated by using DPPH and CUPRAC assays as well as linoleic acid oxidation system, hemoglobin oxidation, and PARP-1 inhibition ELISA. Cardioprotective effect of the defatted dabai peel extract was evaluated using hypercholesterolemic-induced New Zealand white rabbits. Six anthocyanins were detected in the defatted dabai peel, with the highest antioxidant capacities and oxidative stress inhibition effect compared to the other part. The defatted dabai peel extract has also inhibited lipid peroxidation (plasma MDA) and elevated cellular antioxidant enzymes (SOD and GPx) in the tested animal model. Major anthocyanin (cyanidin-3-glucoside) and other anthocyanins (pelargonidin-3-glucoside, malvidin-3-glucoside, cyanidin-3-galactoside, cyanidin-3-arabinoside, and peonidin-3-glucoside) detected in the defatted dabai peel are potential future nutraceuticals with promising medicinal properties.

## 1. Introduction

Anthocyanins are pigments contributed to red, purple, and blue colors of many fruits and vegetables. Anthocyanins have important roles in plant metabolism, such as phytoprotective agents and pollination attractants [1]. As a member of phenolic compounds, anthocyanin is potential antioxidant. Due to their chemical structures, anthocyanins are considered strong antioxidants. Anthocyanin has been reported to actively scavenge free radicals [2] and acts as a potent cardioprotective agent [3].

Dabai (*Canarium odontophyllum*) is a type of olive-like purple-colored fruit that is rich in anthocyanin. Dabai trees can be found in Sumatra, Borneo (Sarawak, Sabah, and East Kalimantan), and Philippines. Chew et al. [4] reported that dabai fruit contained cyanidin-3-glucoside as the major anthocyanin. The anthocyanin is predominantly found in the peel of dabai fruit. Besides dabai, berries such as bilberry, black berry, chokeberry, mulberry, and dogwood berry have high anthocyanin contents. Most of these berries have been found to contain high amounts of cyanidin-3-glucoside [5–7].

Extraction of fat from dabai fruit results in purplish defatted dabai powder that is rich in anthocyanins. During this process, small amount of anthocyanin could be lost in the extracted oil, but most anthocyanins are retained in the defatted dabai powder, especially its peel. Anthocyanins in the defatted dabai powder have great potential in reducing *in vivo* oxidative stress and further prevent cardiovascular diseases. Antiatherosclerotic effects of defatted and nondefatted dabai pericarp and peel have been determined previously in hypercholesterolemic rabbits [8]. Results from several *in vitro* antioxidant assays have also shown protective effect of dabai pericarp [9, 10]. Several biological oxidation activity assays, such as linoleic acid oxidation, hemoglobin oxidation, and PARP-1 inhibition activity, as well as lipid peroxidation marker (plasma MDA) and antioxidant enzymes (SOD and GPx) in blood are good predictors for inhibition of oxidative stress and cardioprotective effect. Therefore, these markers are useful in provision of information and confirmation of the cardioprotective effect of anthocyanins extracted from defatted dabai powder, especially from its peel.

Due to the potential health benefits offered by anthocyanins in defatted dabai, it is of great interest to elucidate the specific anthocyanins in the defatted dabai, especially in its peel. Solid phase extraction (SPE) is a separation technique that had been widely used for purification of polyphenols [11, 12] and pesticides [13]. However, no previous study has been done to fractionate potential anthocyanins in defatted dabai extract using this separation technique. Therefore, this study aimed to determine the anthocyanins content in the extracts and extract fractions of defatted dabai peel and pericarp, their antioxidant capacity, and related-health benefits using *in vitro* assays (DPPH assay, copper (II) reduction antioxidant capacity (CUPRAC) assay, linoleic acid oxidation system, hemoglobin oxidation, and PARP-1 inhibition ELISA). *In vivo* study to evaluate cardioprotective effect of the defatted dabai peel extract on antioxidant enzymes (SOD and GPx) and lipid peroxidation parameter (plasma MDA) of hypercholesterolemic-induced New Zealand white rabbits was also carried out to support the beneficial effect of the extract.

## 2. Materials and Methods

### 2.1. Sample Preparation

Fresh dabai fruits were obtained from a few locations from fruit plantations in Kapit, Sarawak, Malaysia. A homogenized sample was selected randomly from a few different trees in each location. Kernel of dabai fruit was discarded, while dabai peel and a mixture of its pericarp were collected. Both of the samples were freeze-dried using a freeze dryer (Virtis, New York, USA), and the lyophilized samples were ground into smaller particles using a household grinder. To prepare anthocyanin-rich defatted samples, the freeze-dried peel and pericarp were initially defatted by soaking in hexane for 24 h. The defatted samples were redried and further ground into powder using the grinder.

### 2.2. Sample Extraction and Fractionation

Anthocyanins from the defatted dabai powders were extracted based on the optimized conditions that were previously established. Briefly, anthocyanins in the defatted dabai peel and pericarp powders were extracted based on the optimized extraction parameters (53% methanol as solvent and sonicated for 1 min) [14]. The extraction parameters have been optimized previously based on response surface methodology, where the optimized extraction parameters had yielded optimal levels of total phenolics and antioxidant capacity. The mixture was filtered and anthocyanin-rich crude extract was collected. The sample residue was reextracted twice using equal amount of 53% methanol. Methanol was removed using a rotary evaporator (Buchi, Switzerland) at 39°C. The leftover water component and methanol residue of the extracts were fully removed by freeze-drying and stored at −40°C until further analyses.

To obtain a purified anthocyanins fraction, 5.0 mg of the lyophilized anthocyanin-rich crude extract was dissolved with 80% methanol (2 mL, v/v) and fractionated by SPE using activated Sep-Pak CN and C18 cartridges from Merck (Darmstadt, Germany). Visiprep SPE vacuum manifold (12 ports) (Supelco, Pennsylvania, USA) was connected to a vacuum pump and the extracts were fractionated using the cartridges containing 100 mg adsorbent by passing methanol and distilled water (2 mL each) through the cartridges. Methanol fractions were collected but water fractions were discarded. The collected methanolic fractions together with the crude extracts of defatted dabai peel and pericarp were determined for anthocyanins contents and antioxidant capacity together with their oxidative stress inhibition ability.

**Figure 1:**
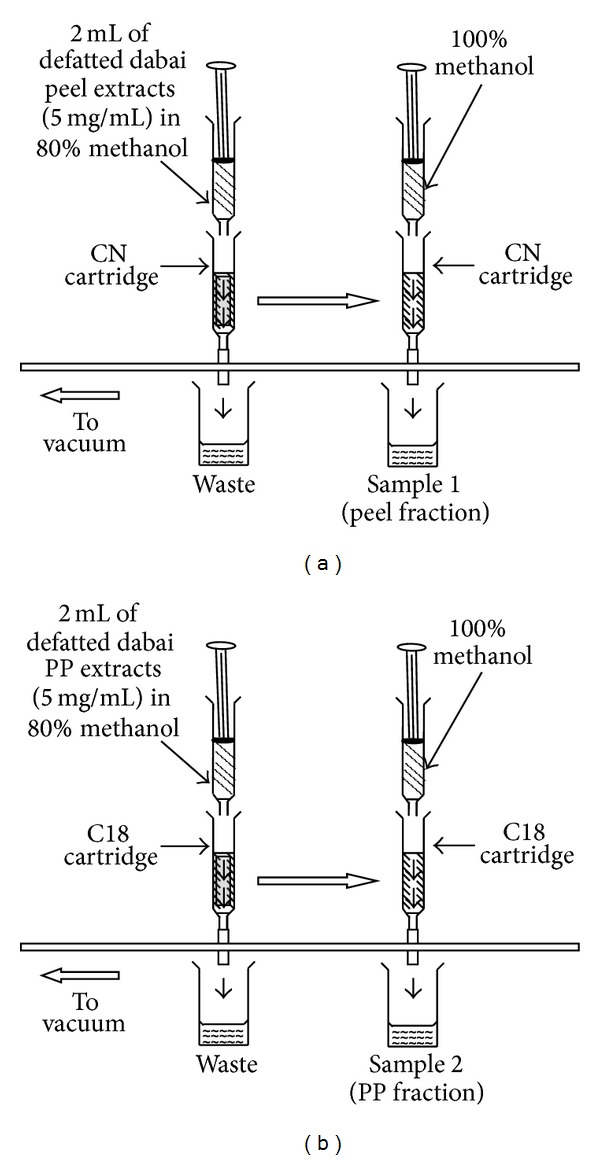
Flow diagrams of sample fractionation [15].

The flow of samples fractionation is shown in Figure 1. Crude extracts of defatted dabai peel and pericarp (2 mL injection volume, 5 mg/mL extract concentration) were injected into the activated Sep-Pak CN and C18 cartridges, respectively, using 5 mL syringes [15]. The adsorbed anthocyanins in both cartridges were eluted by passing 2.0 mL of methanol through the CN cartridge (Figure 1(a)) (for defatted dabai peel) and C18 cartridge (Figure 1(b)) (for defatted dabai pericarp) and collected as samples 1 and 2, respectively.

### 2.3. HPLC Analysis

Anthocyanins in the defatted dabai samples were identified and quantified based on a modified method described by Chew et al. [4]. In this study, UHPLC method using an Agilent 1290 Infinity LC system (Agilent Technologies, Waldbronn, Germany) equipped with a binary pump, diode array detector, and an autosampler was applied in the quantification of anthocyanins. Separation of anthocyanins was achieved on a Lichrospher C-18 column (250 × 4 mm i.d., 5 **μ**m) (Merck KGaA, Darmstadt, Germany) at 30°C. The mobile phases consisted of A: 0.5% trifluoroacetic acid and B: 100% acetonitrile. The gradient was achieved using the following system: 90–85% B, 0–5 min; 85% B, 5–10 min; 85–70% B, 10–15 min; 70% B, 15–20 min; 70–20% B, 20–25 min; 20–0%, 25–30 min. The column was washed and reconditioned after every run. The flow rate was set at 0.6 mL/min and the injection volume was 20 **μ**L.

Anthocyanins were detected at a wavelength of 530 nm, while UV-Vis absorption spectra were recorded in the range of 250–600 nm. A total of six anthocyanin standards, cyanidin-3-glucoside (C3G), cyanidin-3-galactoside (C3L), cyanidin-3-arabinoside (C3A), pelargonidin-3-glucoside (L3G), malvidin-3-glucoside (M3G), and peonidin-3-glucoside (P3G), were used for identification of anthocyanins in the defatted dabai extracts. The anthocyanins content of the defatted dabai extracts was determined and calculated using cyanidin-3-rutinoside (C3R) as internal standard. This is because C3R was not found in all samples. The calculation of anthocyanin content was based on the following equation as suggested by Willis [17]:

(1) Anthocyanin  content  (mg/g  extract) =(weight  of  standard×area  of  anthocyanin×FR   ×100×dilution  factor)  ×(weight  of  sample×area  of  standard)−1,

where

(2) $$\begin{array}{c} F_{R}=\frac{\text{absorbance}/\text{weight}\;\text{for}\;\text{anthocyanin}\;\text{standard}}{\text{absorbance}/\text{weight}\;\text{for}\;\text{anthocyanin}\;\text{sample}}. \end{array}$$

### 2.4. DPPH Radical Scavenging Assay

The antioxidant capacities of anthocyanin-rich crude extracts of defatted dabai peel/pericarp and extract fractions were evaluated by DPPH radical scavenging assay based on the method of Lai et al. [18]. Briefly, aliquots of the anthocyanin-rich crude extracts or extract fractions (0.4 mg/mL) or reference standards (C3G or P3G, 10 **μ**g/mL) were mixed with 0.1 M Tris-HCl buffer (0.8 mL, pH 7.4) and then added to 1.0 mL of 0.5 mM DPPH in methanol. The mixture was vortexed for 10 s and left to stand at room temperature in the dark. After 20 min, the absorbance was measured spectrophotometrically at 517 nm. The antioxidant capacities of the samples were calculated based on the following equation:

(3) Antioxidant  capacity  (%) =[1−absorbance  of  sample  at  517 nmabsorbance  of  control  at  517 nm]×100.

### 2.5. Copper (II) Reduction Antioxidant Capacity (CUPRAC)

The antioxidant capacities of anthocyanin-rich crude extracts of defatted dabai peel/pericarp and extract fractions were measured using copper (II) reduction assay based on a method described by Çelik et al. [19] with some modifications. The anthocyanin-rich crude extracts or extract fractions (0.4 mg/mL) or reference standards (C3G or P3G, 10 **μ**g/mL) (0.1 mL) were mixed with 1.0 mL of 7% methyl-*β*-cyclodextrin solution to completely solubilize the mixture, followed by the addition of 1.0 mL of CuCl_2_, 1.0 mL of copper (II)-neocuproine reagent solution, and 1.0 mL of ammonium acetate solution. The absorbance of final solution (4.1 mL total volume) was measured at 450 nm against a reagent blank after 30 min incubation at room temperature. Calibration curves (absorbance versus concentration graph) of Trolox (0.1–4 mM) were plotted. Trolox equivalent antioxidant capacity (TEAC) was calculated and expressed as mM per gram sample.

### 2.6. Inhibition of Linoleic Acid Oxidation

The inhibition of linoleic acid oxidation was carried out based on a method described by Sultana et al. [20] with some modifications. The diluted anthocyanin-rich crude extracts or extract fractions (0.4 mg/mL) or reference standards (C3G or P3G, 10 **μ**g/mL) (0.2 mL) were added to 0.026 mL of linoleic acid solution, 2.0 mL of methanol (80% v/v), and 1.0 mL of 0.2 M sodium phosphate buffer (pH 7). Then the mixture was made up to 4.0 mL by adding methanol (80% v/v) and incubated at 40°C for 24 h. The inhibition of oxidation was measured as peroxide value applying thiocyanate method [21].

For thiocyanate method, a reacting mixture (0.1 mL) was added with 10 mL of methanol (80% v/v), 0.1 mL of aqueous solution of ammonium thiocyanate (30% w/v), and 0.1 mL of FeCl_2_ solution (20 mM in 3.5% HCl; v/v). After 3 min of stirring, the absorption was measured at 500 nm. Reference standards (C3G or P3G) were used as positive control. The protective capacities (%) of the samples were calculated by applying the following equation:

(4) Protective  capacity  (%)   =[1−Absorbance  increase  of  sample  at  24 hrAbsorbance  increase  of  control  at  24 hr]×100.

### 2.7. Hemoglobin Oxidation

The hemoglobin oxidation was carried out based on a method described by Rodríguez et al. [22] with some modifications. The approval of experimental protocol involving blood withdrawal from rats' tails was granted from the Animal Care and Use Committee of Faculty of Medicine and Health Sciences, Universiti Putra Malaysia (UPM), Selangor, Malaysia (Approval no.: UPM/FPSK/PADS/BR-UUH/00384). Fresh blood was drawn from a group of ten healthy male Sprague-Dawley rats in EDTA-containing tubes. Plasma, platelets, and buffy coat were removed by centrifugation for 10 min at 1853 ×g at 4°C. Red blood cells (RBCs) were washed with PBS two times.

After second wash, the packed RBCs were resuspended gently with PBS to obtain a 5% haematocrit. The RBCs were preincubated at 37°C for 10 min in the presence of 1 mM NaN_3_ (to inhibit catalase activity). Various aliquots of RBCs (1.6 mL) were added to test tubes for each sample treatment. All the test tubes, except the controls tubes, were added with or without the anthocyanin-rich crude extracts or extract fractions (0.4 mg/mL) or reference standards (C3G or P3G, 10 **μ**g/mL). After 24 h of incubation at 37°C, the reacting mixtures were placed in an ice bath for 60 s and centrifuged at 1853 ×g for 10 min at 4°C. The supernatants were collected and determined for TBARS values resulting from autooxidation.

To measure the TBARS, 1.0 mL of the peroxidation product was placed in glass test tube and mixed with 2.0 mL of TCA-TBA-HCl mixture (15% TCA; 0.375% TBA; 0.25 N of HCl) for 10 s using a vortex mixer. The mixture was placed in boiling water for 15 min and left to cool at room temperature for 10 min. The mixture was centrifuged at 1000 ×g for 10 min. The absorbance of the supernatant was read at 535 nm against blank. The protective capacities (%) of the samples were calculated based on the following equation:

(5) Protective  capacity  (%) =[1−Absorbance  increase  of  sample  at  24 hrAbsorbance  increase  of  control  at  24 hr]×100.

The TBARS values of the samples were calculated using a molar extinction of 1.56 × 10^5^ M^−1^ cm^−1^. The results were expressed as TBARS value and calculated as follows [23]:

(6) TBARS  value  (mM) =AbsorbanceMolar  extinction×length  of  cuvette(1 cm).

### 2.8. PARP-1 Inhibition ELISA

The ability of the anthocyanin-rich samples to inhibit PARP-1 activity was determined using an inhibition assay as described by Geraets et al. [24] with slight modifications. Briefly, human rPARP-1 (400 ng/mL) was incubated with a reaction mixture containing 50 **μ**M *β*-NAD^+^ (biotinylated *β*-NAD^+^), 1 mM 1,4-dithiothreitol (DTT), 1.25 **μ**g/mL nicked DNA, and anthocyanin-rich crude extracts or extract fractions (0.4 mg/mL) or reference standards (C3G or P3G, 10 **μ**g/mL) at 4°C. Nicked DNA was prepared by incubating calf thymus DNA with 10 ng/mL DNase I at 37°C for 40 min. The plate was seeded with rPARP-1 overnight (8–12 h) at 4°C before addition of the reaction mixture.

After incubations with the reaction mixture, the plates were washed, and the formation of poly(ADP-ribose)-polymers was detected after 1 h incubation at room temperature with peroxidase-labeled streptavidin (2 *μ*g/mL, 1 : 500 dilution), followed by a 15 min incubation with 3,3′,5,5′-tetramethylbenzidine (TMB) (0.1 mg/mL) in the presence of hydrogen peroxide (0.3%) at 37°C. The reaction was terminated by addition of 0.75 M HCl, and the absorbance was measured at 450 nm. All measurements were carried out in triplicate.

### 2.9. *In Vivo* Study

A total of 21 male New Zealand white rabbits at age of 8–10 weeks were obtained. The initial body weights (1.5–1.7 kg) of the rabbits were recorded and rabbits were caged individually. After two weeks of acclimatization in the ambient temperature of 28°C, the rabbits were randomly distributed into three groups (*n* = 7 per group): normal diet group (NDG), hypercholesterolemic treatment group (HTG), and hypercholesterolemic control group (HCG), also known as positive control group. In NDG, the rabbits were just given normal basal diet. The rabbits from both HTG and HCG received 20 g of hypercholesterolemic diet (normal basal diet containing 0.5% cholesterol) [25], but HTG was additionally supplemented with 2000 mg/day of defatted dabai peel extract throughout the 8-week experimental period. The dosage of 2000 mg/day had been shown to exhibit protective effect [26].

High cholesterol diets were prepared based on 0.5% of total cholesterol in a daily diet, where 0.6 g of cholesterol was dissolved in 2 mL chloroform, and the cholesterol solution was sprayed on the pellets of the rabbits. The pellets were dried in an oven (Memmert GmbH, Schwabach, Germany) at 40°C overnight to allow evaporation of the chloroform [27].

At baseline (week 0) and week 8 of the study, 10 mL of blood was drawn from marginal ear of the fasting rabbits (12 h of fasting). The blood was collected into EDTA-containing tubes for malondialdehyde (MDA) determination, while it was collected in tubes containing lithium heparin for the determination of superoxide dismutase (SOD) and glutathione peroxidase (GPx). The blood samples were centrifuged at 1000 ×g for 10 min at 4°C using a centrifuge (Universal 32, Hettich Zentrifugen, Germany) to separate erythrocytes from the blood's plasma.

MDA levels of the EDTA containing plasmas from HTG and HCG rabbits were evaluated using the TBARS method [23]. The SOD activity of blood samples collected from the rabbits was determined by RANSOD kit (Randox Laboratories, Crumlin, UK) using a Vitalab Selectra Analyzer (Merck, Darmstadt, Germany) [28]. Briefly, the erythrocytes collected were washed four times with 3 mL of 0.9% NaCl solution by centrifugation at 1000 ×g for 10 min. The packed erythrocytes were made up to 2 mL by adding cold distilled water, vortexed for 10 s, and left to stand at 4°C for 15 min. The lysate was diluted with 0.01 mol/L phosphate buffer at pH 7 and mixed thoroughly. The absorbance of the mixture was read at 505 nm. The GPx activity was determined based on the oxidation of glutathione. The oxidized glutathione is immediately converted to the reduced form with the oxidation of NADPH. A blood sample was prepared by diluting 0.05 mL of the whole blood with 2 mL of the diluting agent. RANSEL kit (Randox Laboratories, Crumlin, UK) was used to determine the GPx activity of the blood samples [28]. The decrease in absorbance was measured at 340 nm using the Vitalab Selectra Analyzer.

### 2.10. Statistical Analysis

Anthocyanin contents and antioxidant capacities of the defatted dabai peel and pericarp crude extracts and their SPE fractions were expressed as mean ± standard deviation. Data were subjected to analysis of variance (ANOVA) using a statistical software Minitab version 15. Data for the *in vivo* study were analyzed using (Statistical Package Social Sciences SPSS) version 20.0 (SPSS Corporation, Chicago, IL) for Windows. The differences in group means were determined using one-way ANOVA. Tukey post hoc test was used for multiple group comparison. The level of significance was set at *P* < 0.05.

## 3. Results and Discussion

### 3.1. Anthocyanins in Defatted Dabai Samples

**Figure 2:**
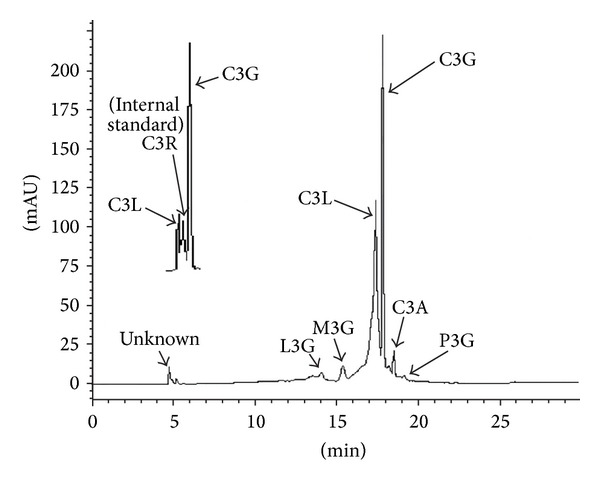
UHPLC chromatogram for the defatted dabai peel extract. See text for the full names of anthocyanins.

Six anthocyanins were identified in the defatted dabai peel. They were L3G, M3G, C3L, C3G, C3A, and P3G. These anthocyanins were eluted from 14.0 min to 19.1 min (Table 1). The RSD (%) of the samples analyzed was lower than 20%. The concentration of each anthocyanin identified in the defatted dabai peel extract was linear (*R* ^2^ = 0.953–0.999). The values for LOD and LOQ of the defatted dabai peel extract are shown in Table 1. The UHPLC chromatogram for the defatted dabai peel is shown in Figure 2.

The peel of purple fruit, such as dabai has high anthocyanin content. Anthocyanin, especially C3G, is the major antioxidant compound in many purple fruits. The result showed that defatted dabai peel had the highest amount of C3G (55.12 ± 0.82 mg/g) compared to other anthocyanins identified. The amount of other anthocyanins in the defatted dabai peel includes C3L (4.42 ± 0.08 mg/g), C3A (4.17 ± 0.27 mg/g), M3G (5.61 ± 0.22 mg/g), and L3G (4.42 ± 0.08 mg/g). Trace amount (below LOQ) of P3G was detected in the defatted dabai peel. In the defatted dabai pericarp, L3G, M3G, and P3G were not detected (below LOD), while trace amount of C3A was found. C3G and C3L in the defatted dabai pericarp were at 6.07 ± 0.02 and 5.84 ± 0.13 mg/g extract, respectively.

SPE had been used to concentrate and purify various crude extracts [29, 30]. However, SPE has not been used in purifying anthocyanin from dabai. In this study, methanolic fractions of the defatted dabai peel and pericarp were collected. These fractions were CN-methanolic fraction of the defatted dabai peel (sample 1) and C18-methanolic fraction of the defatted dabai pericarp (sample 2). The results obtained from our previous study revealed that samples 1 and 2 had the highest total phenolic contents and total anthocyanin contents [15]. Therefore, samples 1 and 2 were considered as the best anthocyanin-rich fractions of the defatted dabai parts if compared to the other extract fractions.

The results obtained from UHPLC analysis revealed that the SPE fractions of the defatted dabai peel had higher amount of C3L compared to the crude extract of the defatted dabai peel. This indicates that SPE method could be able to concentrate anthocyanin compounds such as C3L. The C3G content determined in the SPE fraction (sample 1) of the defatted dabai peel was ~4 times lower compared to the crude extract of defatted dabai peel; while, the amount of C3L found in sample 1 was about four times higher than in the defatted dabai peel crude extract. Trace amounts of L3G, M3G, and P3G were detected in sample 1. The SPE fraction of the defatted dabai pericarp (sample 2) had lower amounts of C3G (4.32 ± 0.17 mg/g) and C3L (4.72 ± 0.07 mg/g) as compared to the crude extract of defatted dabai pericarp. Trace amounts of M3G and P3G were detected in sample 2, while L3G was not detected in the sample. A higher amount of C3A (0.47 ± 0.01 mg/g) was found in sample 2 as compared to the level in defatted dabai pericarp crude extract.

Major anthocyanins found in the defatted dabai samples were C3G, C3L, and C3A. These anthocyanins were also found in many other purple-colored fruits [7, 31]. Although anthocyanins are highly available in blackcurrant and most berries, the anthocyanin, especially C3G, in the defatted dabai peel is a valuable source of potent antioxidant. Moreover, the defatted dabai peel is a by-product of dabai oil extraction that potentially uses as new source of nutraceutical.

Generally, the synthesis of anthocyanins in plant is genetically controlled [32] and regulated initially by phenylalanine ammonia lyase. Phenylalanine as the precursor for anthocyanin synthesis has been reported to be regulated by several enzymes until synthesis of various stable anthocyanidin glycosides [33]. Cyanidin glycoside is the major bioactive compound found in the defatted dabai peel, where naringenin chalcone is known as the main precursor of cyanidin synthesis. Previously, Chew et al. [4] and Khoo et al. [34] reported that naringenin was not detected in dabai fruit. This shows that during fruit ripening, most of the naringenin had been used up for synthesis of cyanidin.

**Figure 3:**
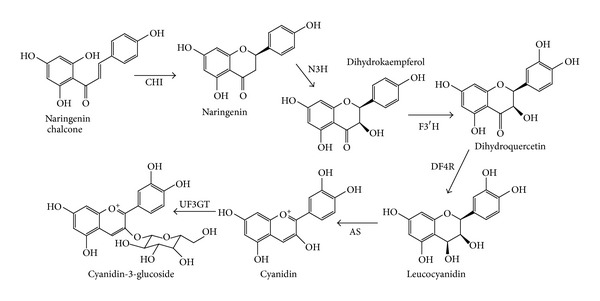
Biosynthetic pathway of cyanidin-3-glucoside from naringenin chalcone in defatted dabai peel [16]. See text for the full names of the various enzymes.

In the C3G synthesis, specific gene regulates naringenin-chalcone synthase to produce naringenin chalcone. Several enzymes have been known to be involved in the synthesis of C3G, such as chalcone isomerise (CHI) and naringenin 3-hydrolase (N3H). The biosynthesis pathway of C3G is shown in Figure 3. The flavanone naringenin is hydrolyzed by N3H into dihydrokaempferol and further hydroxylated to dihydroquercetin by flavonoid 3′-hydroxylase (F3′H) [16, 32]. The dihydrokaempferol is then converted to leucocyanidin by dihydroflavonol 4-reductase (DF4R). During fruit ripening, leucocyanidin is readily converted to cyanidin by anthocyanidin synthase (AS). Due to the unstable form of anthocyanidins, uridine diphosphate glucose flavonoid 3-glucosyltransferase (UF3GT) is readily catalyzed the unstable form of anthocyanidin to a stable anthocyanin [35].

The crude extracts and extract fractions of the defatted dabai parts also contained a trace to undetectable amount of anthocyanidins, where the HPLC peaks of anthocyanidins were below quantitation and detection limits [32]. During extraction, degradation of some anthocyanins, anthocyanidins, and other phenolic compounds might occur. Exposure to light, high temperature, and storage duration are the possible factors for the degradation of anthocyanidins [36]. Besides, degradation of anthocyanins may be due to activity of enzyme dihydroflavonol 4-reductase [37].

### 3.2. Antioxidant Capacity and Inhibition of Oxidative Stress

**Figure 4:**
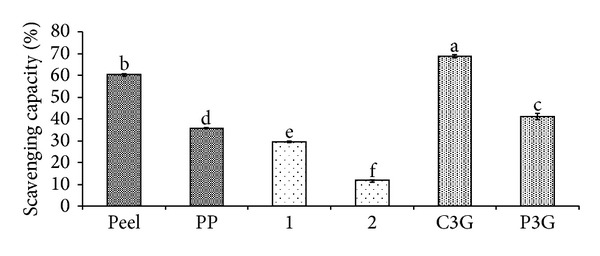
Percentage of scavenging capacities of the anthocyanin-rich extracts, extract fractions, and anthocyanin standards based on DPPH assay. Peel is referring to the defatted dabai peel extract; PP is referring to the pericarp extract of defatted dabai. See text for the full names of anthocyanins.

**Figure 5:**
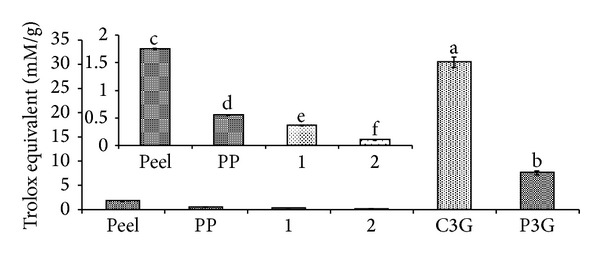
Trolox equivalent antioxidant capacities of the anthocyanin-rich extracts, extract fractions, and anthocyanin standards based on CUPRAC assay.

Anthocyanins in the defatted dabai parts (peel and pericarp) are potential source of nutraceutical. *In vitro *antioxidant assays were used to determine the free radical scavenging effects of the anthocyanin-rich crude extracts of defatted dabai parts. The crude extracts of the defatted dabai peel showed high antioxidant capacities, which were also the highest among the studied samples (Figures 4 and 5). Besides, the crude extracts of defatted dabai parts contained anthocyanins as the major bioactive compounds. For antioxidant assays, 0.4 mg/mL of crude extract or extract fractions and 0.01 mg/mL of anthocyanin standards (C3G and P3G) were tested for their antioxidant capacities and inhibitions of oxidative stress. Our initial screening has shown that 0.4 mg/mL of the crude extracts of defatted dabai peel gave the optimum antioxidant activity. The standards were used for comparison purpose.

The antioxidant capacities of the defatted dabai parts (peel and pericarp) based on chemical assays are shown in Figures 4 and 5. The results showed that the crude extract of defatted dabai peel had significantly highest (*P* < 0.05) antioxidant capacity based on DPPH and CUPRAC assays as compared to other samples. Other samples such as the defatted dabai pericarp extract and their extract fractions (samples 1 and 2) had lower antioxidant capacities.

As assessed using DPPH assay, samples 1 and 2 had the least percentages of scavenging capacities (Figure 4) compared to C3G and P3G. These samples had scavenging capacity less than 50%, except for the crude extract of defatted dabai peel which had 60% of scavenging capacity. The scavenging capacities for both C3G and P3G were 69% and 41%, respectively. The results obtained from CUPRAC assay revealed similar trend of antioxidant capacity as found for DPPH assay. TE values of the anthocyanin standards were the highest (30.42 mM for C3G and 7.61 mM for P3G), followed by the crude extract of defatted dabai peel (1.75 mM) (Figure 5). Based on these results, the crude extract of defatted dabai peel had antioxidant capacity of 4–17 times lower than the anthocyanin standards. This observation suggests that the crude extract of defatted dabai peel is a potentially strong antioxidant, where it mainly consists of anthocyanins (C3G comprised of 75% of the total anthocyanins) (Table 1). As compared with the defatted dabai extracts, lower antioxidant capacities observed for the SPE fractions are possibly due to the presence of other polyphenolics and saponins [15].

The oxidative stress inhibition effect of the anthocyanin-rich crude extracts and extract fractions of defatted dabai was also studied. The effects were determined based on three assays mimicking human vascular system (linoleic acid oxidation system assay, hemoglobin oxidation assay, and PARP-1 inhibition ELISA). In these assays, the same concentration of the extract was applied as in DPPH and CUPRAC assays. Overall, the results showed that the crude extract of defatted dabai peel is a potential nutraceutical for inhibition of oxidative stress. Among the studied samples, the crude extract of defatted dabai peel showed the highest percentages of protective capacity and inhibition activity. On the other hand, other phytochemicals such as flavonoids and saponins have also been detected in the peel and pericarp of dabai fruit [15], where saponin derivative is being one of the major compounds in the defatted dabai pulp. These phytochemicals could have contributed to the protective effects.

**Figure 6:**
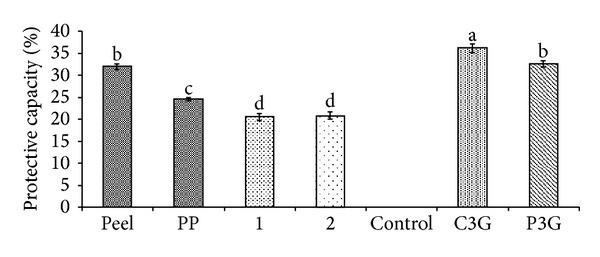
Percentage of scavenging capacity of the anthocyanin-rich extracts, extract fractions and anthocyanin standards based on linoleic acid oxidation system.

**Figure 7:**
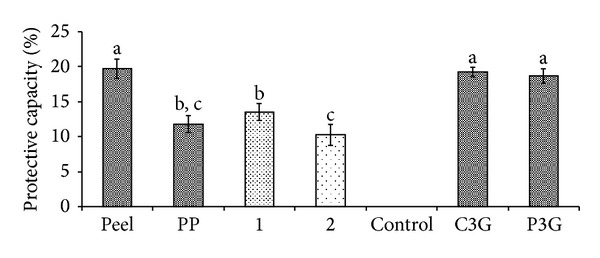
Percentage of protective capacity of the anthocyanin-rich extracts, extract fractions, and anthocyanin standards based on measurement of conjugated diene.

The results from linoleic acid oxidation system assay revealed that samples 1 and 2 had low protective capacity, which were at ~20% (Figure 6). The crude extract of defatted dabai peel showed 32% of the protective capacity, which was significantly higher than the rest of the studied samples. As compared to defatted dabai peel, the pericarp crude extract had low percentage of protective capacity. A possible reason for the low protective capacity is that the defatted dabai peel has high anthocyanins, while the defatted dabai pericarp consists more of the nonanthocyanin compounds, especially from the defatted portion of dabai pulp. Using hemoglobin oxidation assay, concentration of the samples used was low (Figure 7) and was not able to sufficiently result in stronger protective effect in the *in vitro* human vascular model tested.

Based on the linear curve of the crude extract of defatted dabai peel (Table 1), it can be postulated that ~100 times higher extract concentration is needed to give a 50% of protective capacity to the RBC solution. Therefore, we suggest that 1 mg/mL of anthocyanin standards (C3G or P3G) should be able to result in 50% protective capacity in the hemoglobin oxidation system. Besides, a lot of water soluble anthocyanins (L3G and M3G) was also found based on UHPLC analysis as some of the hydrophilic compounds from the extract had been removed by SPE (Table 1). Therefore, the crude extract of the defatted dabai peel could be the best source of nutraceutical for inhibition of oxidative stress.

Generally, the percentages of protective capacity of the crude extracts and extract fractions of the defatted dabai peel and pericarp determined using linoleic acid (Figure 6) and hemoglobin oxidation (Figure 7) assays were less than 50% as compared to the DPPH (Figure 4) and CUPRAC (Figure 5) assays. Comparing the similar extract concentration of defatted dabai peel for both DPPH and linoleic acid oxidation assays, there was about 30% difference in the percentage of inhibition activity. One possible explanation for the difference is that DPPH assay was involved in electron-transfer (ET) reaction pathway, while lipid oxidation assays were involved in hydrogen atom transfer (HAT) reaction pathway. Huang et al. [38] reported that, in ET-based assay, antioxidant acts as a reducing agent, but HAT-based assay involves a radical chain-breaking reaction.

**Figure 8:**
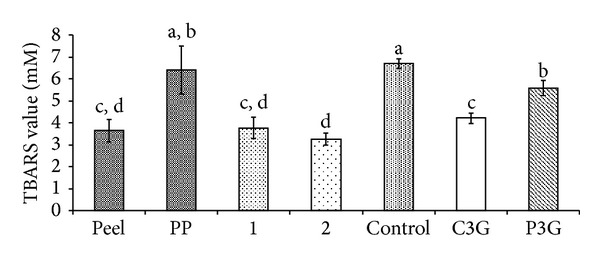
TBARS value of the anthocyanin-rich extracts, extract fractions, and anthocyanin standards based on hemoglobin oxidation assay.

On the other hand, the anthocyanin-rich fraction of the defatted dabai peel showed a low TBARS value based on the hemoglobin oxidation assay (Figure 8). Similarly, the methanolic fractions of defatted dabai peel (sample 1) and pericarp (sample 2) obtained from the SPE also had low TBARS values. Besides, all samples had significantly lower TBARS values than the control (*P* < 0.05), except for the defatted dabai pericarp extract. The standard C3G had TBARS value lower than P3G as assessed by hemoglobin oxidation assay, while the protective capacity (%) and inhibition activity (%) of the standard C3G were higher than the P3G, and the protective capacities of the C3G and P3G were not significantly different (*P* ≥ 0.05). Similar observation has been reported by Kähkönen and Heinonen [39] where low concentration of C3G had higher antioxidant activity than P3G based on LDL oxidation assay. They also found that at higher concentration, P3G showed a stronger antioxidant activity compared to C3G.

**Figure 9:**
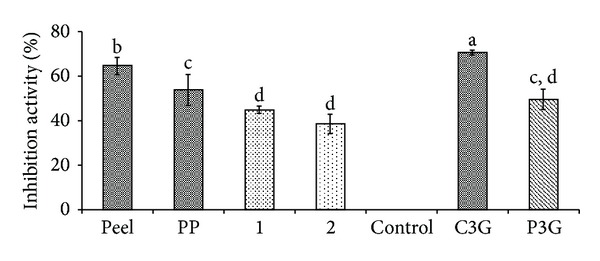
PARP-1 inhibition activity of the anthocyanin-rich extracts, extract fractions, and anthocyanin standards based on PARP-1 inhibition ELISA.

For the PARP-1 inhibition ELISA, all samples showed similar inhibition trends as found for the hemoglobin oxidation assay (Figure 9). Among the samples, crude extract of defatted dabai peel had the highest percentage of inhibition activity (65%), while samples 1 and 2 had the least percentages of inhibition activity (<50%). A comparable inhibition activity was found for both of the crude extract of defatted dabai peel and anthocyanin standards. The high percentage of inhibition activity for the defatted dabai crude extract (65%) and C3G (70%) suggested that C3G in the peel extract was able to inhibit production of PARP-1 compared to P3G (50%) and the other samples which is in agreement with the results obtained based on the linoleic acid oxidation and hemoglobin oxidation assays. Therefore, C3G as the major anthocyanin in defatted dabai peel can be one of the best nutraceuticals for inhibition of oxidative stress.

Previous literatures have consistently shown that anthocyanins possess strong antioxidant activities [40, 41]. Saint-Cricq de Gaulejac et al. [42] postulated that the negatively charged free radicals are prone to react with positively charged flavylium ions and thus prevent oxidation of cellular lipids. As reported by Rice-Evans et al. [43], phenolic compounds with *o*-dihydroxy structure in the B ring have the highest antioxidant activities, while flavonoid with additional hydroxyl group in the B ring has increasing antioxidant activity. Similar to catechin, cyanidin has *o*-dihydroxy structure in the B ring. Therefore, it has high antioxidant activity. Among anthocyanidins, delphinidin has additional hydroxyl group in the B ring. Kähkönen and Heinonen [39] revealed that delphinidin has significantly higher DPPH radical scavenging activity (42%) compared to cyanidin (33%). Chew et al. [4] also reported that delphinidin was detected in dabai fruit obtained from different locations, in which the concentration of delphinidin ranged from not detected to 0.11 mg/100 g dry weight. Although delphinidin-3-glucoside was not determined in the defatted dabai extract, the occurrence of delphinidin in the extract could have contributed to high antioxidant activity. Previous study has also reported that most of the anthocyanin pigments are unstable under neutral or alkaline pH [44]. In this study, the DPPH assay was performed at neutral pH. As observed, anthocyanins such as C3G in neutral pH have shown stronger antioxidant capacity compared to P3G. The possible explanation is that the flavylium cation of C3G formed at low pH may not be a crucial factor for the expression of antioxidative activity, and C3G has been shown as a strong antioxidant at neutral pH [45]. Besides, Kähkönen and Heinonen [39] also showed a significant difference in antioxidant activity for C3G and C3L.

In general, the moderately high antioxidant capacity and protective capacity found for the defatted dabai peel extract were mainly because C3G was the major compound. However, the actual antioxidative mechanism of C3G still remains unknown. Previous studies have reported that exposure of C3G to epithelial cells upregulated nitric oxide (NO) synthase activity by phosphorylation of the enzyme [46, 47]. This is because NO functions as vasodilator [48] and upregulates intracellular cGMP [49] that prevents transportation of calcium into endothelial cells and reduces intracellular calcium concentrations. Increased level of cellular NO has been proven to possess protective effect against vascular damage [50]. Bell and Gochenaur [51] also reported anthocyanin as potent ROS scavenger that could enhance NO-mediated vasorelaxation. Hence, C3G has protective effect against DNA damage by inhibition of xanthine oxidase activity [52].

On the other hand, Hoe and Siong [53] reported that dabai fruit contained 7 mg copper and 1.3 mg iron per 100 g edible portion. These small amounts of trace mineral might affect the antioxidant capacity of dabai extract in CUPRAC and hemoglobin oxidation assays. In the CUPRAC assay, Cu^2+^ from the CUPRAC reagent receives an electron from antioxidant, and the Cu (II) is reduced to Cu (I) [54]. Trace amount of copper ions from dabai extract might also receive electrons from the antioxidant in the extract, and the copper ion from the extract possibly acted as chromogen in the CUPRAC assay. Thus, these copper ions could have contributed to some inconsistency in the results obtained. Similarly, trace iron ions found in dabai extract could affect the results obtained from hemoglobin oxidation assay. In nonstress condition, heme ion in hemoglobin normally remains unaffected. Under oxidative stress condition, heme ion from the hemoglobin is released and coupled with the ions from the extract which has high possibility to further induce oxidative stress in the hemoglobin solution. Addition of antioxidant in the reacting mixture might yield inaccuracy and inconsistency in the results obtained. These metal ions and heme compounds have also been found to decompose lipid peroxidase [55], where decrease in peroxidase activity enhances oxidative stress. Therefore, further study on hemoglobin oxidation assay needs to consider the degree of oxidative stress induced to hemoglobin.

### 3.3. Cardioprotective Effect of Defatted Dabai Peel

To further strengthen the beneficial effect observed using *in vitro* systems, an *in vivo* study using the defatted dabai peel extract was performed. As compared to baseline values, significant increase of the plasma MDA levels was detected for both HCG and HTG at week 8 of high cholesterol diet. In HCG, the plasma MDA increased about 1.44 mol/L (~50%) after 8 weeks of the study. As compared to NDG, 8 weeks of normal chow diet has resulted in elevation of plasma MDA levels by about 16% (1.28 mol/L) which indicate the normal physiological oxidation of the *in vivo* system. However, a significantly low increment (0.19 mol/L) of plasma MDA was found for the HTG after 8 weeks of supplementation with the anthocyanin-rich extract of defatted dabai peel (Table 2) as compared to the HCG.

From baseline to 8 weeks of the study, the HCG had a significant increase in SOD activity. The result also showed that 8-week supplementation of the defatted dabai peel extract (HTG) resulted in significant reduction of SOD activity (35.6%) compared to the HCG, while the GPx levels for both HTG and HCG were significantly reduced compared to baseline after 8 weeks of the study. High cholesterol diet given to the rabbit for 8 weeks has been demonstrated to significantly increase the SOD activity (42%) and inhibit the GPx activity (18%) as compared to the NDG (Table 2). Excessive dietary cholesterol has enhanced oxidative stress in the hypercholesterolemic rabbits, thus increased the cellular SOD activity. Similarly, oxidative stress is known to accelerate production of MDA in cellular system of the rabbits. High cholesterol diet in the experimental rabbits has also exacerbated the MDA production in both HTG and HCG.

MDA is a secondary lipid peroxidation product, while SOD is an enzyme that catalyzes two molecules of superoxide radicals to hydrogen peroxide and oxygen in order to reduce the oxidative stress in cellular level. Increased cellular MDA and SOD indicate acceleration of oxidative stress level initiated by free radicals. High levels of MDA and SOD are good indicators of oxidative stress as the increasing level of MDA indicates the acceleration of lipid peroxidation, while high SOD activity shows the high level of superoxide radicals in the cellular system. In this study, reduction of MDA and SOD is a good indicator of cardioprotection.

On the other hand, the increase in GPx activity may not show cardioprotective effect. It is due to the involvement of GPx enzymes in breaking down of hydrogen peroxide that has been produced by SOD in high oxidative stress condition. The result of this study demonstrated that the no significant change of GPx activity between HTG and HCG does not imply any cardioprotective effect of the defatted dabai peel extract as the activity of GPx is an adaptation to the abundant activity of SOD in the system. GPx enzyme is one of the indicators for monitoring oxidative stress instead of indicating the reduction of oxidative stress. Besides, hydrogen peroxide in the cellular level should have been catalyzed or converted to water and oxygen by potential catalase and antioxidants [56].

Previous study has shown that 5% supplementation of defatted dabai powder was able to improve plasma lipid profile of hypercholesterolemic rabbits [25]. The cholesterol lowering effect of the defatted dabai powder has proven its protective effect in hypercholesterolemic condition [8, 25]. In this study, the use of defatted dabai peel extract at 2000 mg/day has successfully reduced oxidative stress condition in the hypercholesterolemic rabbits. We have also confirmed that the defatted dabai peel extract used contains >70% of anthocyanin. Based on this *in vivo* study, we are able to show that about 150 mg of the total anthocyanins in the defatted dabai powder would protect an *in vivo* system from excessive damage due to the oxidative stress condition.

Several reported benefits of anthocyanins have further strengthened the protective effect of defatted dabai peel extract. Anthocyanin-rich potato flake has significantly improved the antioxidant status in the serum of hypercholesterolemic rats treated with the flake (300 g/kg diet) [57]. Besides, anthocyanin-rich juice extracts of berries have shown to be antihyperlipidemic in the hypercholesterolemic animals [58, 59]. Conversely, anthocyanin-rich extract and juice from blackcurrant had no improvement in plasma lipid profile in hyperlipidemic rabbits [60], thus suggesting that anthocyanins are somehow prooxidant. However, the results from our study demonstrated the ability of anthocyanins from defatted dabai peel for cardioprotection. Besides MDA and SOD, as indicators for CVD, cellular oxidized LDL is also one of the risk factors for DNA damage.

As oxidative stress plays an important role in DNA damage, DNA damages induced by oxidative stress coupled with LDL oxidation were closely related to atherosclerosis [61]. Kong et al. [62] also suggested possible defence mechanism for protection of anthocyanin against oxidative DNA damage, where copigmentation of cyanidin-DNA has helped to reduce DNA damage. Besides NO synthase, heme oxygenase-1 could be one of the possible protective mechanisms for C3G in endothelial dysfunction [63]. Cellular NAD^+^ and PARP-1 are other parameters that are closely related to oxidative stress. During oxidative stress, activation of PARP-1 is closely linked to reduction in NAD^+^ [64]. El-Mahdy et al. [65] reported that naringenin was found to inhibit activation of PARP-1 cleavage. Similar to naringenin, the catechol structure of anthocyanin is also able to show protective effect against oxidative stress through inhibition of PARP-1 activation. Thus anthocyanin could help in reduction of rapid depletion of cellular NAD^+^ during high stress condition.

## 4. Conclusions

Anthocyanins in the defatted dabai peel are potential antioxidants. In the crude extract of defatted dabai peel, C3G was the major anthocyanin with stronger antioxidative properties compared to P3G. C3G also acted as reducing agent and powerful free radical scavenger as determined by DPPH and CUPRAC assays. C3G also possessed higher oxidative stress inhibition effects through inhibitions of linoleic acid oxidation, hemoglobin oxidation, and PARP-1 activity compared to the other anthocyanins. Higher amount of C3G was also obtained from the extract fraction of defatted dabai peel as compared to the extract fraction of the pericarp. The results of *in vitro* assays had also proven that the C3G-rich extract of defatted dabai peel is a potential cardioprotective agent. The protective effect was further supported by *in vivo* study, where the C3G-rich extract of the defatted dabai peel has inhibited MDA production in the hypercholesterolemic rabbits, and able to maintain the physiological regulation between the oxidant and the antioxidant in the *in vivo* system.

## Figures and Tables

**Table 1:** Anthocyanins content of the defatted dabai extracts and extracts fractions and other UHPLC parameters.

Sample	Anthocyanins (mg/g extract)					
	Pelargonidin-3-glucoside	Malvidin-3-glucoside	Cyanidin-3-galactoside	Cyanidin-3-glucoside	Cyanidin-3-arabinoside	Peonidin-3-glucoside
Defatted dabai peel extract	4.42 ± 0.08	5.61 ± 0.22	4.42 ± 0.08	55.12 ± 0.82	4.17 ± 0.27	Trace
Defatted dabai pericarp extract	ND	ND	5.84 ± 0.13	6.07 ± 0.02	Trace	ND
1	Trace	Trace	17.45 ± 0.4	13.46 ± 0.28	1.08 ± 0.03	Trace
2	ND	Trace	4.72 ± 0.07	4.32 ± 0.17	0.47 ± 0.01	Trace
Other parameters						
Retention time	14.0 ± 0.2	15.3 ± 0.3	17.3 ± 0.4	17.8 ± 0.2	18.4 ± 0.5	19.1 ± 0.3
RSD (%) for all samples	1.77–3.22	1.27–4.63	1.56–9.98	0.14–3.96	0.25–6.78	0.42–15.49
RSD (%) for defatted dabai peel	1.77	4.0	1.77	1.48	6.58	9.01
Linearity (*R* ^2^) for defatted dabai peel	0.953	0.978	0.953	0.999	0.989	0.963
LOD for defatted dabai peel (mg/mL)	0.27	1.15	0.28	0.31	0.01	0.82
LOQ for defatted dabai peel (mg/mL)	1.23	5.15	4.68	1.38	0.03	3.68

*ND: not detected; LOD: limit of detection; LOQ: limit of quantitation. See text for the names of samples 1 and 2.

**Table 2:** MDA levels, SOD, and GPx activities of the blood samples from *in vivo* study.

Groups	Plasma MDA (mol/L)		SOD (U/mL)		GPx (U/mL)	
	Baseline	Week 8	Baseline	Week 8	Baseline	Week 8
NDG	2.39 ± 0.08	3.67 ± 0.05	4.80 ± 0.13	2.94 ± 0.09	0.76 ± 0.05	0.71 ± 0.09
HTG	2.70 ± 0.07	2.89 ± 0.09	4.73 ± 0.09	3.24 ± 0.12	0.78 ± 0.08	0.56 ± 0.12
HCG	2.83 ± 0.12	4.27 ± 0.14	4.77 ± 0.09	5.03 ± 0.11	0.81 ± 0.06	0.59 ± 0.09

NDG: normal diet normal, HTG: hypercholesterolemic treatment group, and HCG: hypercholesterolemic control group.
